# The newcomer’s guide to health technology assessment: a collection of resources for early career professionals

**DOI:** 10.1017/S0266462324000424

**Published:** 2024-11-04

**Authors:** Antonio Migliore, Debjani Mueller, Wija Oortwijn

**Affiliations:** 1 Health Technology Assessment international (HTAi), Edmonton, AB, Canada; 2 Department of Public Health, University of Pretoria, Hatfield, South Africa; 3 IQ Health Science Department, Radboud University Medical Center, Nijmegen, The Netherlands

Capacity building for health technology assessment (HTA) holds a pivotal position in shaping the landscape of healthcare decision-making. It is one of the key strategic goals of Health Technology Assessment International (HTAi) for the development and use of HTA around the globe. Over the past decade, HTAi has initiated several activities to define capacity building for HTA (1;2). This endeavor underscores the multifaceted nature of capacity building within HTA, encompassing the development or enhancement of competencies crucial for understanding, contributing to, executing, or leveraging HTA for health policy formulation and decision-making. Moreover, capacity building extends to fostering awareness and gathering support within the broader ecosystem where HTA operates (3).

A few years ago, the required competencies for the conduct of HTA were published (3). Competencies required within the HTA field span a wide spectrum of knowledge, skills, and attitudes, reflecting the multidisciplinary essence of HTA and its application context and ideally covering the entire health technology lifecycle (4).

However, recruiting organizations that employ HTA professionals face a unique challenge, as the knowledge and skill sets of candidates often do not align with the multifaceted demands of their roles (5). HTA master’s programs offering comprehensive training in the required competencies of HTA practitioners are increasing but still limited in quantity and geographical reach (3). The HTAi Educational Offers Database (6) currently includes nine master’s programs from different parts of the world. Acknowledging this reality, it becomes evident that for individuals embarking on the HTA journey, supplemental educational resources still play a crucial role in augmenting HTA knowledge and skills not typically covered in conventional study programs, such as patient and public involvement or ethical, legal, and socio-cultural aspects.

In response to this need, the HTAi Early Career Network initiated in 2017 the development of a repository of educational content specifically tailored to newcomers in the HTA field. This ambitious initiative engaged several contributors and reviewers from within HTAi’s diverse membership, including Interest Groups, Committees, and the Board of Directors. The resultant collection of open-access resources serves as a toolbox for navigating the intricate landscape of HTA, offering resources ranging from introductory primers to in-depth methodologies and processes. Importantly, these resources complement the capacity-building initiatives coordinated and offered by the Society to the broader community.

Each resource within this curated collection is designed to be easily accessible, providing links to externally published materials such as handbooks, guidelines, scientific articles, and conference proceedings. While these resources are not a substitute for comprehensive training in HTA conduct, they do lay the groundwork for understanding the main HTA principles, tools, methods, and outputs.

To truly excel in the field of HTA, newcomers are encouraged to pursue accredited education and training programs and adhere to specific guidelines. It is crucial to perceive the HTAi Newcomers’ Guide to HTA as a dynamic repository – a “living collection” – where new resources can be continually incorporated to enrich its coverage and utility in facilitating the growth and proficiency of HTA professionals. So far eight resources are part of the collection: *HTA 101: Essential Information for Newcomers* presents a basic introduction to HTA, while other resources are more specific and focus on particular aspects of HTA such as writing a research protocol, evaluating ethical aspects, hospital-based HTA, patient and social engagement, issues on publication, disseminating and implementing HTA findings, involving health professionals and economic evaluation. The collection, which can be found online on the HTAi portal (7), continues to seek additional resources to cover specific needs of the HTA community in order to promote the development and use of HTA around the globe.
